# Neonatal Brain Injury and Neuroanatomy of Memory Processing following Very Preterm Birth in Adulthood: An fMRI Study

**DOI:** 10.1371/journal.pone.0034858

**Published:** 2012-04-20

**Authors:** Anastasia K. Kalpakidou, Matthew P. Allin, Muriel Walshe, Vincent Giampietro, Kie-woo Nam, Philip McGuire, Larry Rifkin, Robin M. Murray, Chiara Nosarti

**Affiliations:** 1 Department of Psychosis Studies, Institute of Psychiatry, King's Health Partners, King's College London, London, United Kingdom; 2 Department of Neuroimaging, Institute of Psychiatry, King's Health Partners, King's College London, London, United Kingdom; Beijing Normal University, Beijing, China

## Abstract

Altered functional neuroanatomy of high-order cognitive processing has been described in very preterm individuals (born before 33 weeks of gestation; VPT) compared to controls in childhood and adolescence. However, VPT birth may be accompanied by different types of adverse neonatal events and associated brain injury, the severity of which may have differential effects on brain development and subsequent neurodevelopmental outcome. We conducted a functional magnetic resonance imaging (fMRI) study to investigate how differing degrees of neonatal brain injury, detected by neonatal ultrasounds, affect the functional neuroanatomy of memory processing in VPT young adults. We used a verbal paired associates learning task, consisting of four encoding, four cued-recall and four baseline condition blocks. To further investigate whether differences in neural activation between the groups were modulated by structural brain changes, structural MRI data were also collected. We studied 12 VPT young adults with a history of periventricular haemorrhage with associated ventricular dilatation, 17 VPT individuals with a history of uncomplicated periventricular haemorrhage, 12 individuals with normal ultrasonographic findings, and 17 controls. Results of a linear trend analysis demonstrated that during completion of the paired associates learning task right frontal and right parietal brain activation decreased as the severity of neonatal brain injury increased. There were no statistically significant between-group differences in on-line task performance and participants' intelligence quotient (IQ) at assessment. This pattern of differential activation across the groups was observed particularly in the right middle frontal gyrus during encoding and in the right posterior cingulate gyrus during recall. Structural MRI data analysis revealed that grey matter volume in the right superior temporal gyrus, right cerebellum, left middle temporal gyrus, right globus pallidus and right medial frontal gyrus decreased with increasing severity of neonatal brain injury. However, the significant between-group functional neuroanatomical differences were not directly attributable to the detected structural regional differences.

## Introduction

Children and adolescents who were born before 33 weeks' gestation (very preterm; VPT) show poorer performance compared to controls on a variety of memory tasks, including working, spatial and episodic memory in childhood and adolescence [1]–[4].

Damage to the hippocampus, possibly due to hypoxia-ischemia, has been postulated to underlie memory deficits in VPT populations [3], [5], [6]. However, only a few studies to date have explored the functional neuroanatomy of mnemonic processing in VPT individuals. Curtis and colleagues [7] reported alterations in VPT children compared to controls in the caudate nucleus during completion of a spatial working memory task, while Gimenez and colleagues [8] described increased activation in right hippocampus during the encoding of novel face-name pairs in VPT adolescents. Our group previously demonstrated altered fronto-temporal activation in VPT young adults during performance of a verbal paired associates learning task, and fronto-parieto-occipital alterations during performance of a visual version of this task [9], [10]. These studies investigated heterogeneous groups of VPT individuals [7], [8], or excluded individuals with severe brain injury [9], [10]. Since VPT birth may be accompanied by different types of adverse neonatal events and associated brain damage, it would be important to study early events in relation to adult neuroanatomical changes.

The most common form of brain injury following VPT birth is periventricular haemorrhage (PVH), which is well-recognized on neonatal cranial ultrasounds [11]. PVH may occur either in isolation (i.e. Uncomplicated PVH – UPVH), when confided to the germinal matrix, or may be concomitant with ventricular dilatation (VD; PVH+VD), following extension of the haemorrhage in the lateral ventricles [12].

The greatest degree of neonatal insult, i.e. PVH+VD, is likely to cause the greatest disruptions in brain growth in VPT individuals [13], [14]. VPT children with PVH+VD were found to have reduced subcortical grey matter compared to their VPT peers without PVH+VD [15]. In adolescence, VPT individuals with PVH+VD exhibited more numerous cortical and subcortical structural alterations compared to VPT individuals with UPVH and those with normal ultrasound results, with differences being noted in frontal and temporal cortices, thalamus, corpus callosum and cerebellum [16]. Impaired cortical development following UPVH in VPT infants has also been described [17].

In addition, individuals who experienced PVH+VD are the most vulnerable to developmental compromise [18]. For instance, PVH+VD was associated with increased risk of deficits in visual associative learning in school-aged VPT children [19], and with lower IQ and increased behavioural problems in VPT adolescents [20]. The impact of UPVH on the neurodevelopment of VPT individuals has been a matter of debate [21], however, there is evidence that even UPVH may exert some deleterious effect on specific aspects of neurodevelopment, such as memory and language functions [22]–[24].

To our knowledge, no study to date has investigated whether the functional neuroanatomy of memory processing in VPT individuals varies according to their degree of neonatal brain injury. Verbal paired associates learning tasks are used to assess the episodic memory processes [25]–[27], which are implicated in the formation of new memory traces (encoding processes), in the maintenance of these memory traces over time (storage processes), and in supporting access to stored memory traces at a later time (retrieval processes), the means of which are recognition and recall [28]. In comparison to storage processes, which are temporarily distributed, encoding and retrieval processes happen at specific time points and are therefore well suited to be studied with fMRI [29].

We used fMRI with a verbal paired associates learning task we previously used [9], [30] in four groups of participants with different degrees of severity of neonatal brain injury or no brain injury: (1) VPT young adults with a history of PVH+VD; (2) VPT young adults with a history of UPVH; (3) VPT young adults with no history of neonatal brain injury; (4) term-born controls. In the current study, encoding of paired associates was studied for pairs of words and retrieval was assessed by the production of words to specific cues (cued-recall) [31]. As both verbal encoding and recall processes are mediated by fronto-temporo-parietal cortices [27], [32]–[34], we hypothesized that there would be differential activation of this network in VPT individuals with differing degrees of neonatal brain injury [16]. We predicted that greater functional neuroanatomical alterations would be associated with increasing severity of neonatal brain injury. We further analyzed structural MRI data to investigate the association between functional neuroanatomical alterations and potential differences in brain structure [9].

## Methods

### Ethics Statement

Ethical approval for the study was granted by King's College Hospital Research Ethics Committee. All participants gave their written informed consent to undergo assessments.

### Participants

Between 1979 and 1984, 368 infants born very preterm (<33 gestational weeks) were admitted to the neonatal unit at the University College London Hospital (UCHL), and survived to be discharged. All VPT individuals received neonatal ultrasound scans daily for the first 4 days, at 1 week, and weekly until discharge from the hospital [11]. A linear-array ultrasound scanner (ADR 2130) equipped with a 5 or 7 MHz probe was used to perform the scans. The images were either stored on videotapes or as Polaroid photographs. These infants were all enrolled for participation in longitudinal follow-up studies [35]–[37].

At 14–15 years, 269 individuals of the original cohort agreed to be assessed. Results of the adolescent assessment have been previously published [16], [38], [39]. At age 20 years, 94 individuals of those assessed in adolescence underwent further neuropsychological assessment [40]. A sub-sample of these individuals participated in a series of fMRI studies [9], [10], [41], [42].

The current study included 19 of those VPT individuals who had previously participated in fMRI studies and 22 newly–recruited VPT individuals. VPT study participants were chosen on the basis of their neonatal ultrasonographic findings. These were classified according to previously specified criteria [16]: normal neonatal ultrasonographic findings; UPVH: PVH into the germinal layer or ventricles without subsequent ventricular dilatation or parenchymal involvement; PVH and VD: PVH with dilatation of either one or both lateral ventricles. However, the degree of ventricular dilatation was inadequate to meet the diagnostic criteria for hydrocephalus.

All VPT participants were dextral, as assessed by clinical neurological examination at 14–15 years of age. Exclusion criteria were: severe head injury, stroke, epilepsy and multiple sclerosis, severe eyesight impairment, hearing and/or motor impairment, metal implants or a fitted pacemaker, operations to the head or the spine, claustrophobia, and pregnancy for female participants.

Term-born control data were drawn from healthy individuals previously studied and were selected according to age, handedness and gender in order to be comparable to the VPT participants [9], [10], [41], [42]. Exclusion criteria, other than those common to the VPT study participants, were: birth complications (e.g., preterm birth <37 weeks of gestation, low birth weight defined as <2500 grams, endotracheal mechanical ventilation), prolonged gestation (greater than 42 weeks), and history of psychiatric illness.

All participants were English native speakers.

### Sample Characteristics

One VPT participant with a history of psychiatric illness was excluded from the analyses. Twelve VPT young adults with a history of PVH+VD (newly recruited), 17 with a history of UPVH (9 previously studied and 8 newly recruited), 12 VPT young adults with normal ultrasonographic findings (10 previously studied and 2 newly recruited), and 17 term-born controls were studied (all previously studied). Information about sex, age at assessment, educational level and socio-economic status (SES), categorized according to a standard occupational classification [43], was available for all study participants. The four groups did not differ significantly in sex [x^2^_(3)_ = 0.45, p>0.05], SES [x^2^_(6)_ = 5.14, p>0.05] and educational level [x^2^_(9)_ = 12.48, p>0.05], but there were significant between-group differences in age at assessment [F _(3, 54)_ = 9.88, p<0.001]. Post-hoc comparisons showed that the PVH+VD group was significantly older than the normal VPT [Mean difference  = 3.87, p<0.01] and the control groups [Mean difference  = 3.83, p<0.01].

Neonatal data i.e. birth-weight (grams) and gestation at birth (weeks) were previously collected for VPT study participants only. There were no significant differences in birth-weight [F _(2, 38)_ = 1.42, p>0.05] and gestation at birth [F _(2, 38)_ = 0.66, p>0.05] between the three VPT groups.

Table 1 displays descriptive statistics for the neonatal and socio-demographic data.

**Table 1:** Neonatal and socio-demographic data of the study groups.

Variable+	PVH+VD (n = 12)	UPVH (n = 17)	Normal VPT (n = 12)	Controls (n = 17)
Neonatal/socio-demographic characteristics				
Birth-weight (g)	1122.33 (395.60)	1274.47 (396.70)	1388.92 (372.58)	n/a ++
Gestation at birth (weeks)	28.42 (2.64)	28.76 (2.14)	29.5 (2.43)	n/a
Males/Females (number)	7/5	8/9	6/6	8/9
Age (yrs) at assessment *	24.58 (2.48)	22.65 (2.57)	20.69 (1.92)	20.75 (1.37)
Socio-economic status at assessment (number) ^a^				
I – II	6	10	6	7
III	6	5	5	5
IV – V	0	2	1	4
Educational level b				
O-level only	3	2	0	1
A-level or exultant	2	6	7	5
3^rd^ level education	7	9	5	9

+ Mean and standard deviation (SD) are presented, unless otherwise stated.

++ n/a  =  non-applicable *p<0.001, ^a^ For controls n = 1 missing data.

b For controls n = 2 missing data.

### Neuropsychological data

Four subtests from the Wechsler Abbreviated Scale of Intelligence (WASI) [44] (i.e. vocabulary, block design, similarities and matrix reasoning), were used to estimate verbal, performance and full-scale IQ.

### fMRI Task

To examine the neural correlates of paired-associate learning, we used a verbal task based on the Paired Associates Learning subtest of the Wechsler Logical Memory Scale – Revised [45]. This task was used by our group in previous studies [9], [30]. The task involved an encoding, a recall and a ‘fonts’ discrimination condition (baseline), as well as a ‘blanks’ low-level baseline, presented in that order, in a total of 16 blocks of 8 pairs of word-stimuli (four blocks with a total of 32 presentations of word-pairs per condition). All the words used for the task were selected from the MRC Psycholinguistics Database [46] and were matched in number of letters, frequency in the written language and meaningfulness [47]. Each block of word-pairs lasted 40 seconds (s). The inter-stimulus interval i.e. the time between the display onsets of two pairs of stimuli was 5 s and was given by the sum of the duration of the silent period (3.5 s) and the compressed image acquisition (1.5 s). The long inter-stimulus interval was chosen to allow for the longer reaction time latencies of very preterm-born individuals and to provide them with a slightly longer than usual interval of rest between trials [48].

**Figure 1:**
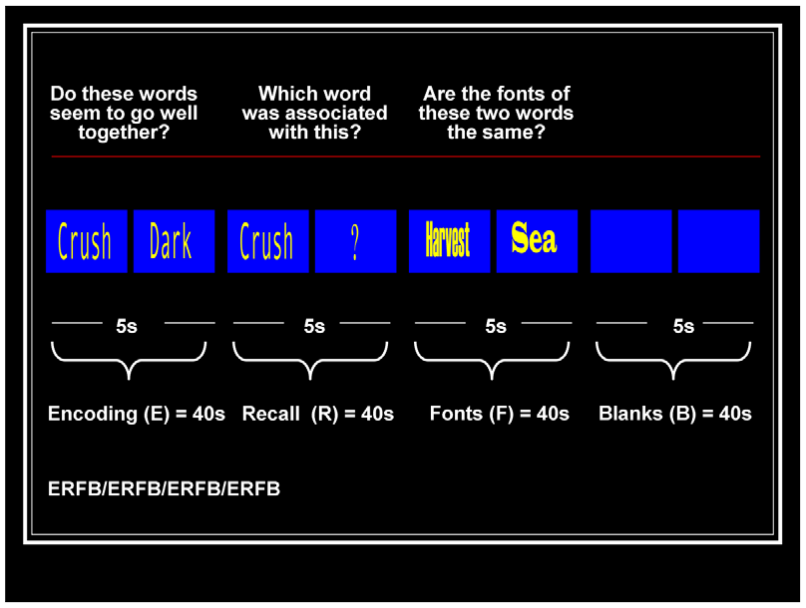
Graphic representation of the verbal paired associates learning paradigm.

The experimental conditions and the baseline were presented 4 times each and are described below (Figure 1): Encoding condition – Participants were visually presented with pairs of nouns written on blue rectangles, and were instructed to verbally say (‘Yes’/‘No’) if they thought the nouns of each pair were associated. The order of the presentation of the word-pairs was randomized across blocks. Recall condition – A single word from each pair previously presented during encoding, was displayed with a question mark and participants were required to verbally say the other word of the pair it had been presented with. On failure to recall the word, participants were instructed to articulate the word ‘pass’. ‘Fonts’ discrimination condition – It required the participants to overtly say (‘Yes’/‘No’) when asked if the fonts of the words of each pair were the same. This condition was designed to control for activation associated to the processing of non-mnemonic information (i.e. reading and semantic processing). An ‘instruction question’ was displayed on the computer screen, prior to the presentation of each encoding (‘Do these words seem to go well together?’), recall (‘Which word was associated with this?’) and ‘fonts’ discrimination condition (‘Are the fonts of these two words the same?’). ‘Blanks’ low-level baseline – Participants were presented with two identical, empty blue rectangles, of the same dimensions as those presented during the encoding and the retrieval conditions and were instructed to simply look at them.

In order to be familiarized with the task, all participants underwent an off-line training session before the fMRI session. Two repetitions of the 4 blocked-conditions were performed, presenting 4 pairs of words per blocked-condition. The words used for the purposes of the training session were different from those presented during scanning.

In the analysis, ‘blanks’ low-level baseline condition was subtracted from all other conditions. In order to isolate mnemonic aspects of encoding and recall processes (and exclude possible effects associated with reading and semantic processing), analysis subtracting the ‘fonts’ discrimination condition (baseline) from encoding and recall conditions was also performed.

### Image acquisition

MR images were obtained using a 1.5 Tesla GE MR Sigma System (GE Medical Systems, Milwaukee, WI, USA) at the Maudsley Hospital, London. For radio frequency transmission and reception, a quadrature birdcage head coil was used.

#### fMRI Acquisition

At each of 16 non-contiguous near axial slices (slice thickness  = 7 mm, gap  = 0.7 mm), 148 T2*-weighted functional images were obtained oriented parallel to the intercommisural (anterior commisure – posterior commisure) plane so that the whole brain would be covered (repetition time – TR  = 1500 ms, echo time – TE  = 40 ms, flip angle  = 70°, 1 excitation, field of view – FOV  = 240^2^ mm, matrix size  = 64^2^ mm, in-plane resolution  = 3.75^2^ mm, scan time  = 735 s).

#### Structural MRI Acquisition

A 43 slice high resolution structural image (slice thickness  = 3 mm, gap  = 0.3 mm, TR  = 3000 ms, TE  = 40 ms, flip angle  = 90°, 8 excitations, FOV  = 240^2^ mm, matrix size  = 128^2^ mm, in-plane resolution  = 1.88^2^ mm, scan time  = 72 s) was also collected and used during the normalization of individual functional data into standard Talairach space.

A 124 slice, three-dimensional T1-weighted gradient-echo image-sequence allowing for slice reconstruction in any plane (slice thickness  = 1.5 mm, TR  = 35 ms, TE  = 5 ms, flip angle  = 35°, 1 excitation, FOV  = 240×180 mm, matrix size  = 256×128 mm, in-plane resolution  = 0.94^2^ mm, scan time  = 434 s) was obtained to facilitate structural brain analysis.

### fMRI data analysis

#### Individual and group brain mapping

The data were analyzed using the XBAM_v4 software developed at the Institute of Psychiatry, King's College London (http://www.brainmap.it) [49]. This non-parametric approach, which allows for p values to be estimated accurately with minimal assumptions, was chosen as the most appropriate given the high likelihood of non-parametric distribution in fMRI data [50]. Data were processed to correct motion, intensity and spin excitation history [50] and were smoothed prior to statistical analysis and normalization, i.e. in native space. The fMRI voxel dimensions were 3.75 mm in-plane and the slice thickness was 7.7 mm. A Gaussian filter of 8.8 mm FWHM was used, which we deemed appropriate, given the resolution of the images and the likely size of the activated regions. Once pre-processing was completed, single subject analyses in native space were performed. The software detected and modelled blood-oxygen-level dependent (BOLD) responses to each experimental condition using Gamma variate functions (peak responses at 4 and 8 seconds). The sum of squares (SSQ) ratio, a goodness-of-fit statistic was then computed at each voxel. This consisted of the ratio of the sum of squares of deviations from the mean intensity of the image due to the model (model time series) to the sum of squares of deviations due to the residuals (original time series minus model time series). The data were then permuted using a wavelet-based method, which allowed the calculation of the null distribution of SSQ ratios assuming no experimentally determined response [51]. Individual brain activation maps for each individual for each condition of the task were computed. To reduce the possible confounding effects of differential task performance between the groups on BOLD signal, in each recall block of 8 responses each, only activation related to correct responses was modelled. For instance, if the second and fourth verbal pair was incorrectly recalled, the average recall activation for that block was made up of six rather than 8 stimulus pairs (the model in this example would have estimated 1 0 1 0 1 1 1 1 rather than 1 1 1 1 1 1 1 1). Prior to group analysis, the observed and the randomized SSQ ratio maps (statistical maps) from each individual were transformed into a standard stereotactic space [52]. This was a two-stage procedure; the statistical maps were first realigned to the same individual's high resolution structural image and were then normalized to a Talairach template [49]. The Talairach template currently used in XBAM was produced using the Talairach transformation facility of Analysis of Functional Neuroimages (AFNI) software for analysis and visualization of functional magnetic resonance neuroimages [53]. Once the individual statistical maps were in Talairach space, group brain activation maps were computed for each task condition. The median of the observed and randomized SSQ ratio maps over all individuals at each voxel was calculated. The distribution of the median of the randomized SSQ ratio maps was then used to obtain the null distribution of SSQ ratios.

#### Group comparison

In order to identify brain regions which were differentially activated across the study groups, analysis of variance (ANOVA) was used, testing for a linear trend in regional brain activation where PVH+VD < UPVH < normal VPT < controls and where PVH+VD > UPVH > normal VPT > controls. This model fitted the data at each intracerebral voxel at which all individuals had non-zero data. In order to reduce outlier effects, the model was fitted by minimizing the sum of absolute deviations, rather than the sum of squares. For the computation of the null distribution, group comparison data were permuted under the assumption of no condition or group effect, followed by refitting of the above model. Group comparison maps were produced using XBAM cluster analysis. This is a two-stage procedure using a preliminary voxel level threshold of 0.05 to maximise sensitivity (minimise type II errors), followed by a cluster level threshold, which is chosen to control type I cluster errors at whole brain level. As participants' age at assessment ranged from 20.75 to 24.58 years, age was used as a covariate in the analyses.

SSQ values were extracted from cluster local maxima where differential activation across study groups was evident, in order to be used for graphical representation of the data in the results section. Labels for brain regions with activation local maxima were determined using Talairach coordinates in all stages of the data analysis.

### Structural MRI data processing and analysis

The three-dimensional structural MRI data sets were processed using voxel-based morphometry in Statistical Parametric Mapping SPM8 (Wellcome Department of Cognitive Neurology, Institute of Neurology, London, UK, http://www.fil.ion.ucl.ac.uspm/software/spm8), running on Matlab 7.8 (Math-Works, Natick, USA). Initially, images underwent pre-processing consisting of the following stages: 1. Each T1-weighted image was affined-registered into the SPM T1 template and was segmented into different tissue types (i.e. grey and white matter); 2. the affined-registered grey matter images were used to create a customized template using the DARTEL algorithm [54]; 3. The affined-registered grey matter images were normalized to the customized DARTEL template and were modulated for non-linear components. After the pre-processing, quality check of grey matter segmentation was performed via a sample homogeneity check. Grey matter images were then smoothed with a 12 mm Gausian kernel and used for subsequent statistical analysis.

A linear trend whole-brain analysis was performed to examine grey matter volume differences across the four study groups (PVH+VD < UPVH < normal VPT < controls and PVH+VD > UPVH > normal VPT > controls). Grey matter eigenvalues for each study participant were calculated for each cluster where significant between-group differences were observed; SPM's ‘volume of interest’ data extraction tool was used. Structural volume coordinates were originally reported in Montreal Neurological Institute (MNI) space. They were then converted to Talairach space using a Java applet, which employs the Nonlinear Yale MNI to Talairach Conversion Algorithm (www.bioimagesuite.org) [55].

### Statistical analysis of non-imaging data

Statistical analyses were carried out with SPSS v15.0 (SPSS, Chicago, USA). To explore sex, SES and educational level distribution across study groups, a chi-square test for independence (x^2^) was used. Group comparisons in terms of age at assessment, neonatal and neuropsychological data and on-line behavioural measures (those acquired during completion of the fMRI tasks), were performed using one-way univariate ANOVA. Between-group differences in age at assessment were further investigated with post-hoc comparisons using a Games-Howell test. To test the effect of learning on study participants' performance across the four cued-recall blocks of the verbal paired associates learning task, a one-way repeated measures ANOVA was used.

To explore the link between birth-weight and functional data, multiple linear regression analysis was carried out. We used the SSQ values extracted from all regions where differential activation across PVH+VD, UPVH and normal VPT groups and controls were observed, as dependent variables. Birth-weight in grams was entered as predictor in the analysis.

In order to investigate the relationship between structural and functional data, multiple linear regression analyses were performed using the SSQ values extracted from all regions where between-group differences were observed, as dependent variables, and grey matter eigenvalues extracted from all regions where structural between-group differences were found, as predictors.

## Results

### Neuropsychological performance

ANCOVA, controlling for age at assessment, revealed no statistically significant differences in full scale [F _(3, 50)_ = 1.78, p>0.05], verbal [F _(3, 50)_ = 1.60, p>0.05] and performance [F _(3, 50)_ = 1.39, p>0.05] IQ between the PVH+VD, UPVH and normal VPT groups and controls.

Table 2 displays descriptive statistics for neuropsychological data of the study groups.

**Table 2:** Neuropsychological and on-line behavioural data of the study groups.

Variable+	PVH+VD (n = 12)	UPVH (n = 17)	Normal VPT (n = 12)	Controls (n = 17)
Neuropsychological performance (WASI) a				
Full-scale IQ	105.92 (6.97)	106.47 (9.77)	97.92 (12.43)	107.71 (13.93)
Verbal IQ	104.75 (10.07)	102.06 (9.62)	95.67 (13.52)	105.29 (12.12)
Performance IQ	106.08 (11.41)	109.06 (10.63)	100.17 (11.26)	108.43 (15)
On-line task performance				
Accuracy (number of correct responses)	27.00 (2.69)	30.35 (2.34)	28.42 (3.73)	28.35 (4.56)

+ Mean and standard deviation (SD) are presented, unless otherwise stated.

a For controls n = 3 missing data.

### On-line task performance

ANOVA revealed that there were no significant between-group differences in the mean number of correct responses given during the recall condition of the task [F _(3, 54)_ = 2.31, p>0.05]. A one-way repeated-measures ANOVA showed a significant effect of learning, measured in terms of number of correctly recalled words in the cued-recall phase of the task across the four blocks, for all study participants [F _(3, 55)_ = 32.21, p<0.001].

Table 2 displays descriptive statistics for on-line task performance of the study groups.

### Functional MRI results

#### Group differences

Linear trend analysis revealed significant differences in regional brain activation across the four study groups, with PVH+VD < UPVH < normal VPT < controls during encoding in the right middle frontal gyrus (Broadmann area – BA 46) and during recall in the right posterior cingulate gyrus (BA 30), after covarying for age at assessment. There were no statistically significant differences for the opposite contrast i.e. PVH+VD > UPVH > normal VPT > controls.

**Figure 2:**
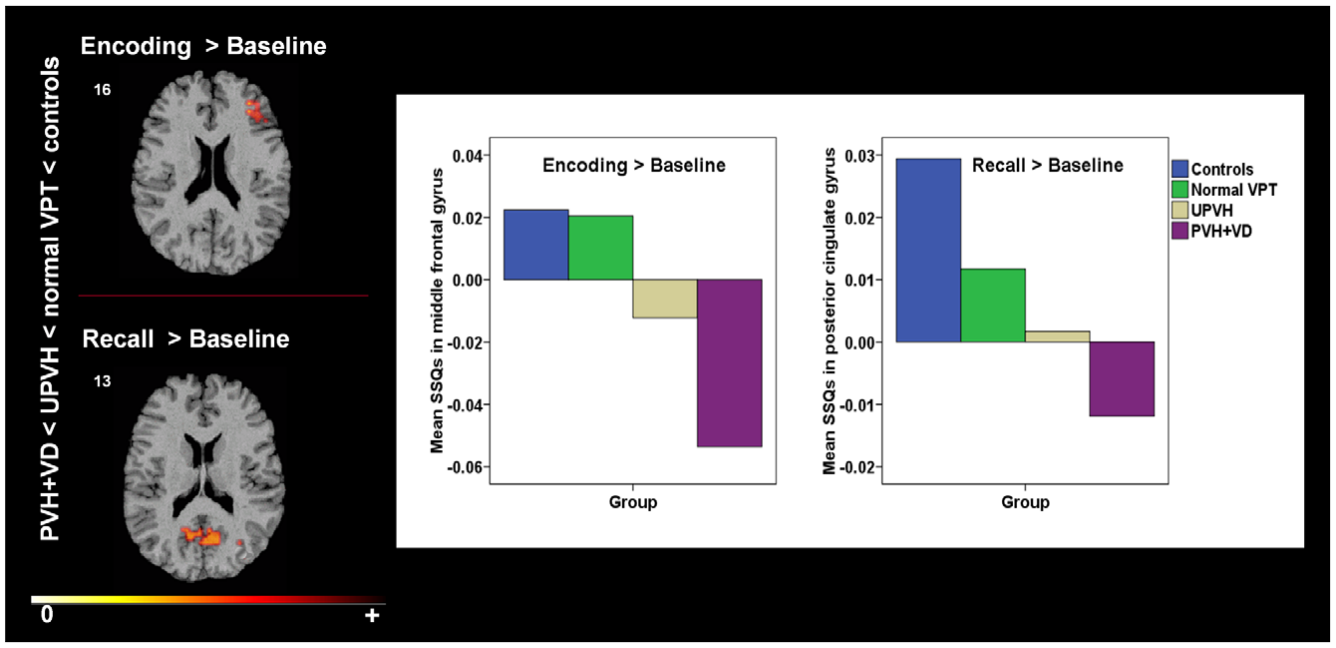
Between-group differences in regional brain activation during a verbal paired associates learning task. Coloured areas signify PVH+VD < UPVH < normal VPT < controls. The numbers at the top of each row of slices represents the z coordinate in Talairach space. The right side of the brain corresponds to the right side of each slice. The graph shows cluster local maxima where differential activation across study groups was observed, as indicated by the mean of SSQ values.

Table 3 shows the 3D clusters detected for between-group differences for each condition. Figure 2 displays brain activation maps for between-group differences, as well as graphical displays of the SSQ values in PVH+VD, UPVH and normal VPT groups and controls, as extracted by cluster local maxima were between-group differences were observed.

**Table 3:** Between-group differences in regional brain activation during a verbal paired associates learning task. a

Task condition	Brain Region (Broadmann area)	Side	Talairach Coordinates (x, y, z)	Cluster size	Cluster p value
PVH+VD < UPVH < normal VPT < controls					
Encoding	Middle frontal gyrus (46) extends: anteriorly to right middle frontal gyrus (10) and inferiorly to inferior frontal gyrus (46)	R	41, 36, 16	28	0.0051
Recall	Posterior cingulate gyrus (30) extends: superiorly to posterior cingulate (31) bilaterally	R	14, −62, 13	53	0.0035

a Results refer to encoding and recall conditions contrasted with ‘fonts discrimination’ condition (baseline).

To explore the relationship between birth-weight and SSQ values in brain regions where functional between-group differences were observed, multiple linear regression analysis was performed. Results suggested that birth-weight did not have a significant unique contribution to the prediction of the SSQ values in the right middle frontal gyrus [F _(1, 39)_ = 0.73, p>0.05], and right posterior cingulate gyrus [F _(1, 39)_ = 0.01, p>0.05].

### Structural brain differences

Linear trend whole-brain analysis showed significant differences between the four groups in grey matter volume i.e. absolute amount of grey matter, in the right superior temporal gyrus (BA 22), right cerebellum, left middle temporal gyrus (BA 21), right globus pallidus and right medial frontal gyrus (BA 6), where PVH+VD < UPVH < normal VPT < controls (see Table 4).

**Table 4:** Between-group differences in grey matter volume.

Brain Region (Broadmann area)	Side	Talairach Coordinates (x, y, z)	Cluster Size	SPM (Z)
Superior temporal gyrus (22) extends to:	R	51, −17, 4	11383	5.38
precentral gyrus (6)	R	53, −5, 9		5.07
middle temporal gyrus (22)	R	53. −12, −8		4.80
Cerebellum	R	20. −41, −11	13092	5.26
Middle temporal gyrus (21)	L	−55, −12, −7	2532	4.87
Globus pallidus extends to:	R	17, −11, −3	7678	4.83
thalamus	R	−7. −8, 16		4.52
Medial frontal gyrus (6)	R	22, 3, 50	1376	4.54

Results are FDR (false discovery rate) corrected p<0.05.

### Structure-function associations

Multiple linear regression results indicated that structural between-group differences i.e. structural grey matter volume in the right superior temporal gyrus (BA 22), right cerebellum, left middle temporal gyrus (BA 21), right globus pallidus and right medial frontal gyrus (PVH+VD < UPVH < normal VPT < controls) were not significantly associated with functional differences in right middle frontal gyrus (BA 46) [F _(5, 52)_ = 1.34, p>0.05] and in right posterior cingulate gyrus (BA 30) [F _(5, 52)_ = 1.14, p>0.05], where PVH+VD < UPVH < normal VPT < controls during encoding and recall, respectively.

## Discussion

This study demonstrates that the adult neuroanatomy of mnemonic processing is modulated by the severity of neonatal brain injury. This occurs in a linear fashion, with those individuals with a history of periventricular haemorhage with associated ventricular dilatation (PVH+VD) displaying the greatest extent of right hypofrontality and decreased right parietal activation during performance of a verbal paired associates learning task compared to the other three groups. In addition, VPT individuals with a history of uncomplicated periventricular haemorrhage (UPVH) show decreased activation compared to VPT individuals with normal ultrasound findings, who, in turn, show reduced activation compared to controls.

The study groups (i.e. PVH+VD, UPVH, normal VPT, controls) displayed a comparable on-line performance and achieved comparable scores on measures of IQ and they did not signicantly differ in terms of educational level at assessment. These data are in line with previous fMRI studies describing altered brain activation in VPT young adults in the absence of significant between-group differences in on-line task performance or measures of IQ at assessment [9], [10], [41].

During encoding, our results identified altered regional brain activation in VPT young adults with differing degrees of neonatal brain injury, where PVH+VD < UPVH < normal VPT < controls, in the right dorsolateral prefrontal cortex (DLPFC), in a cluster with local maxima in right middle frontal gyrus (BA 46), extending ipsilaterally to the middle frontal gyrus (BA 10), and to the inferior frontal gyrus (BA 46). Studies investigating the neuroanatomy underlying the encoding of paired-associates which are subsequently remembered have suggested the involvement of the right DLPFC, possibly due to its role in organizing information in working memory, thereby strengthening associations among items in episodic memory [56]–[59].

During recall, the same pattern of differential activation across the study groups i.e. PVH+VD < UPVH < normal VPT < controls was observed in a cluster with local maxima in the right posterior cingulate gyrus (BA 30), extending bilaterally to posterior cingulate (BA 30 and 31). Lesion studies suggest that damage to the posterior cingulate gyrus may result in loss of verbal episodic memory [60]. Neuroimaging studies also suggest that this brain region is involved in recall processes of verbal episodic information [61]–[63], possibly due to its strong reciprocal connections with the medial temporal lobe [64], [65].

Taken as a whole, the results of the current study show that regional brain activation decreases with increasing severity of neonatal brain injury in regions mediating verbal paired-associate learning (task-specific regions: posterior cingulate gyrus) and more extensively, in regions subserving working memory, an executive component of the verbal paired associates learning task (DLPFC). Hypoactivity in the DLPFC has been described in developmentally delayed populations, such as individuals with attention deficit hyperactivity disorder (ADHD) during executive-type tasks [66], [67].

Nevertheless, in spite of attenuated activation in these regions, we observed similar levels of on-line task performance in VPT young adults with differing degrees of neonatal brain injury and controls. This may be because the studied task was relatively easy to perform, as we wished to ensure all participants would be able to complete it during the scanning session. Behavioural differences may become apparent with increasing cognitive load, when VPT individuals with differing degrees of neonatal brain injury may fail to optimally engage task specific areas and more general ‘executive’ brain areas, which could result in impaired task performance [20], [24], [68]. Non-significant between-group differences in on-line task performance may also be accounted for by a ceiling effect. In addition, it is possible that fMRI techniques are particularly sensitive in detecting differences at the neuronal activation level between small groups of participants, like those included in the current study, whereas behavioural analyses require larger groups to provide reliable results [69], [70]. Differences in neuronal activation between the groups may be partly explained by structural brain changes associated with very preterm birth and neonatal brain injuries [8], [9]. This study investigated participants' structural MRI data, which was analysed in the same way as the fMRI data, i.e. with linear trend analysis. Voxel based morphometry revealed significant between-group differences in grey matter volume in the right superior temporal gyrus, right cerebellum, left middle temporal gyrus, right globus pallidus and right medial frontal gyrus, where the PVH+VD group showed the greatest decreases compared to the other three groups. This finding is consistent with previous studies reporting the greatest alterations in grey matter and white matter volume in VPT individuals with a history of severe neonatal brain injury (i.e. PVH+VD) [16], [38]. Additionally, this pattern of structural abnormalities, which includes the temporal and frontal cortices, the cerebellum and the globus pallidus has been observed in neurodevelopmental disorders such as ADHD [71], [72].

When investigating the relationship between participants' structural volume in the regions listed above where significant between-group differences were observed and functional MRI results, we did not detect a statistically significant association. This is in contrast to previous studies in similar samples, where we observed that structural differences between the groups accounted for a small percentage of the variance of fMRI results [10], [41]. Our current results suggest that in this instance between-group differences in regional brain activation may not be solely interpreted in the context of structural brain alterations [73].

However, we cannot rule out that differences in neuronal activation between the groups may be associated with cytoarchitectonic changes in the brain regions where structural between group differences were observed (as well as in others which are known to be affected by neonatal brain injury) [74], [75], which could not be studied with the methods of structural analysis we used in this study. Preterm birth has in fact been associated with abnormalities in neural architecture and connectivity [76].

A limitation of this study is that the fMRI data reported here may not be generalizable to VPT populations – for example VPT young adults who have compromised cognitive function were not studied. Additionally, we have not collected any data on other factors that may have contributed to fMRI differences among the study groups, such as family history of psychiatric disorders and maltreatment during development. Other limitations include the age difference between VPT participants with a history of neonatal brain injury (PVH+VD, UPVH) and VPT participants with normal ultrasonographic findings and controls. The majority of the participants belonging to these two latter groups (see Methods sections) were scanned at an earlier time point i.e. approximately 5 years before the PVH+VD and UPVH groups, due to logistic reasons. We, however, controlled for age at assessment in the fMRI data analyses. Further limitations arise from the use of a previously scanned normal VPT and control group and relate to possible implications of scanner variation over time. Nevertheless, fMRI experiments measure changes in BOLD signal, which is less affected by hardware changes as it relies on an innate contrast mechanism between experimental conditions and a baseline. Quality control of the fMRI data acquired throughout the time of the two studies was also carried out to identify variations of BOLD signal intensity using an automated data processing scheme and Shewhart charting [77]. Finally, the sole use of a linear trend analysis of the fMRI data acquired during performance of the verbal paired associates learning task could have limited the identification of brain regions where differential activation might have only been present in pair-wise analyses. A similar limitation applies to the analysis of the structural data, which were analyzed using a linear trend approach.

To summarise, the results of this study suggest that increased severity of early brain injury is associated with decreased neural recruitment in regions mediating verbal paired-associate learning (i.e. posterior cingulate gyrus) and in regions postulated to be involved in more general ‘executive-type’ processing (i.e. DLPFC). Although the current study did not detect significant differences in on-line task performance between the groups, the sub-optimal neural engagement of task-specific and of ‘executive’ brain regions in VPT individuals, and especially in those with severe early injury, poses the question whether behavioural differences may become apparent with increasing cognitive load, which will be the focus of future studies.
